# HuoXueJieDu Formula Alleviates Diabetic Retinopathy in Rats by Inhibiting SOCS3-STAT3 and TIMP1-A2M Pathways

**DOI:** 10.1155/2017/4832125

**Published:** 2017-11-29

**Authors:** Hongliang Wang, Wei Xing, Shijie Tang, Zhenglin Wang, Tiantian Lv, Yan Wu, Shuzhen Guo, Chun Li, Jing Han, Ruixin Zhu, Wei Wang

**Affiliations:** ^1^College of Basic Medicine, Key Laboratory of Ministry of Education (Syndromes and Formulas), Key Laboratory of Beijing (Syndromes and Formulas), Beijing University of Chinese medicine, Beijing 100029, China; ^2^School of Public Health, Capital Medical University, Beijing 100069, China; ^3^Key Laboratory of Systems Biology, CAS Center for Excellence in Molecular Cell Science, Innovation Center for Cell Signaling Network, Institute of Biochemistry and Cell Biology, Shanghai Institutes for Biological Sciences, Chinese Academy of Sciences, Shanghai 200032, China; ^4^Institute of Chinese Medicine, Beijing University of Chinese medicine, Beijing 100029, China; ^5^Modern Research Center for Traditional Chinese Medicine, Beijing University of Chinese Medicine, Beijing 100029, China; ^6^Department of Bioinformatics, School of Life Sciences and Technology, Tongji University, Shanghai 200092, China

## Abstract

HuoXueJieDu (HXJD) formula exerts protective effects against diabetic retinopathy (DR) in rats, but its underlying mechanism remains unknown. In the present study, the diabetic rats were established using streptozocin. The administration of HXJD was initiated at 20 weeks after diabetes induction and continued for 12 weeks. Whole genome expression profiles in rat retinas were examined using microarray technology. Differential gene expression and pathway enrichment analysis were conducted on the microarray data, with validation through real-time PCR and immunohistochemical staining. The results showed that 170 genes and several IPA canonical pathways related to inflammation, matrix metabolism, and phototransduction were regulated by HXJD. PCR validation of selected genes, including SOCS3, STAT3, TIMP1, and A2M, confirmed the gene expression changes influenced by HXJD. In addition, the immunohistochemical staining results suggested that critical members of the SOCS3-STAT3 pathway were also affected by HXJD. Taken together, these results indicated that SOCS3-STAT3 and TIMP1-A2M pathways might mediate the alleviation of HXJD activities in rats with diabetic retinopathy.

## 1. Introduction

Diabetic retinopathy (DR) is defined as a retinal disease resulting from diabetes, characterized by microvascular lesions in the retina, including vascular proliferation, vascular leakage, and retinal ischaemia [1]. DR has been attributed to 4.8% of the 37 million blind patients worldwide, and this disease is the most common cause of visual impairment in the working-age population [2]. The number of individuals suffering from DR has increased because diabetes has recently become a global epidemic disease.

Until recently, hyperglycaemia control, laser photocoagulation, and vitreoretinal surgery have been the main treatments for DR. However, the progress of DR in some patients has not been arrested or even reversed by these efforts. Thus, new therapies that could block the development of retinal neovascularization need further exploration.

HuoXueJieDu (HXJD) is a Chinese herbal formula comprising *Euonymus alatus* (Gui-Jian-Yu), *Radix trichosanthis* (Tian-Hua-Fen), *Panax notoginseng* (San-Qi), and *Coptis chinensis* (Huang-Lian). HXJD originates from clinical experience and could stabilize vision and reduce retinal oedema. In addition, previous studies have shown that in the central retinal artery of diabetic rats, HXJD improves peak systolic velocity, end diastolic velocity, and mean velocity and decreases the pulse index and resistance index [3]. In addition, HXJD increases the wave amplitudes (the maximal electric reaction for dark-adapted eyes) and the sum amplitude of the oscillatory potentials (OPs) in the electroretinogram [4]. Furthermore, HXJD reduces vascular density and the ratio of endothelial cells to pericytes in the retinas of diabetic rats [3]. This effect was consistent with the downregulation of apoptosis [5], VEGF (vascular endothelial growth factor) [6], and MMPs (matrix metalloproteinases) [7]. These results indicate that HXJD confers protection against retinal injuries in DR, but the mechanism underlying the effect of HXJD has not been elucidated.

In recent years, high-throughput omics technologies, particularly microarray transcriptomes, have been increasingly applied in traditional Chinese medicine research [8, 9]. Thus, to address the mechanism of HXJD, we assessed the protective effects of this compound on DR in STZ-induced rats, and microarray technology was subsequently used to analyze the influence of HXJD on the gene expression profile. Furthermore, some of the potential targets were validated using PCR. The results of the present study will reveal the biochemical pathways underlying the effect of HXJD and provide further insight into the therapeutic approaches for the prevention of DR.

## 2. Methods

### 2.1. Ethic Statement

All procedures involving the animals and their care were performed according to the governmental guidelines on animal experimentation and the “Principles of Laboratory Animal Care” of the National Institutes of Health. All experimental protocols were approved by the Institutional Animal Ethics Committee of Beijing University of Chinese Medicine, Beijing, China (permit number 26-1514).

### 2.2. Antibodies

The following antibodies were used in the present study: anti-JAK2 antibody (number 3230; Cell Signaling Technology, Boston, Massachusetts, USA); anti-p38 MAPK antibody (ab197348; Abcam, Cambridge, UK); anti-NF-*κ*Bp65 antibody (ab16502; Abcam); anti-phospho-stat3 antibody (number 9145; Cell Signaling Technology, Boston, Massachusetts, USA); anti-STAT3 (number 4904, Cell Signaling Technology, Boston, Massachusetts, USA); anti-TIMP1 (ab61224, Abcam, Cambridge, UK); anti-SOCS3 (ab16030, Abcam, Cambridge, UK); and anti-A2M (ab58703, Abcam, Cambridge, UK).

### 2.3. Plant Materials

*E. alatus* (Gui-Jian-Yu), *R. trichosanthis* (Tian-Hua-Fen), *P. notoginseng* (San-Qi), and *C. chinensis* (Huang-Lian) were obtained from Tongrentang (Beijing, China). Professor Peng Tan (College of Chinese Medicine, Beijing University of Chinese Medicine, Beijing) authenticated the processed Chinese medicinal materials. The plant specimens (Number TCM120305, 120306, 120307, and 120308) were stored at the YIFU Research Building of Beijing University of Chinese Medicine.

### 2.4. Preparation of Plant Extract

*E. alatus*, *R. trichosanthis*, *P. notoginseng*, and *C. chinensis* were extracted for 2 hours under reflux with water. The plants were extracted 2 times, and the extracts were pooled and vacuum concentrated at 60°C.

Ultraviolet spectrophotometry was applied to determine the major component contents in the extracts using rutin as a standard, with a content of 17%. Ginsenoside Rg1 was used to represent the content of saponins at 16%, while berberine represented the typical content of alkaloids at 20%, and glucose was used as the standard to assess the content of polysaccharides at 18%. The extracts were dissolved in water.

### 2.5. Animals

Healthy, male Sprague-Dawley rats (7 weeks of age, 200–250 g) were purchased from Vital River Laboratory Animal Technology Co. Ltd. (Beijing, China, certificate number SCXK (Beijing) 2007-0001). The animals were raised in a clean grade animal laboratory with a temperature of 22–24°C and 40%–60% humidity. The rats were provided commercial rat feed and water *ad libitum*. At the end of the experiments, the animals were sacrificed by intraperitoneal injection with pentobarbital (50 mg/kg). The blood glucose was examined every four weeks.

### 2.6. Induction of Diabetes

Streptozocin (STZ, Sigma, St. Louis, Mo USA, catalog number S0130) was dissolved in citrate buffer (PH4.5) with a concentration of 20 g/L. The dissolved STZ was preserved in the dark. The rats were administered an intraperitoneal injection of STZ at 65 mg/kg body weight [10]. The control rats received equal volumes of citrate buffer. Four weeks later, blood glucose level in a blood sample obtained from the tail vein was evaluated using a standard glucometer (One Touch Profile, LifeScan, San Francisco, California, USA). Rats with blood glucose levels higher than 16.7 mmol/L were selected for subsequent experiments.

### 2.7. Treatment Schedule

Twenty weeks after STZ injection, the diabetic rats were grouped into two groups according to the glucose concentration and body weight: diabetic (*n* = 8) and HXJD (*n* = 8, 15.4 g/kg). Subsequently, the diabetic rats in HXJD group were administered an HXJD decoction through intragastric gavage. The rats in the normal (*n* = 8) and diabetic groups were administered with water. After 12 weeks of treatment, the rats were anaesthetized, and the retinas were removed.

### 2.8. Haematoxylin-Eosin (HE) and Immunohistochemical Staining

The retinas were isolated, fixed in paraformaldehyde, and embedded in paraffin. The paraffin sections were deparaffinized and dehydrated. Subsequently, the sections were stained with haematoxylin-eosin. The thickness of retinas from the photoreceptor layer (PL) to the inner plexiform layer (IPL) in the midretina was determined, and the number of the retinal ganglion cells (RGC) were assessed. For immunohistochemistry, after dewaxing in benzene and dehydration in gradient alcohol, the sections were incubated with 0.3% hydrogen peroxidase to remove endogenous peroxidases. Subsequently, the sections were incubated with primary antibody (dilution of antibody: JAK2 1 : 50, p38 MAPK 1 : 100 and NF-*κ*Bp65 1 : 500) overnight at 4°C. After three washes with PBS, the sections were incubated with secondary antibody at room temperature. Finally, the sections were stained with 3,3′-diaminobenzidine (DAB) and haematoxylin. The sections were visualized using a microscope and analyzed using ImageJ software.

### 2.9. mRNA Microarray Analysis

Total RNA from the retinas of rats was extracted. The integrity of the RNA was assessed using the Agilent Bioanalyzer 2100 (Agilent Technologies, Palo Alto, California, USA). Only samples with RNA integrity values larger than 7 and 28S/18S ratios larger than 0.7 were considered qualified. Subsequently, 100 ng of RNA was amplified, labelled, and purified according to the manufacturer's instructions. After hybridization, the microarrays were washed and scanned with default settings according to the manufacturer's instructions (Agilent Technologies, USA). Background correction, quantile normalization, and differential expression analyses were performed using the limma package in the *R* (version 3.2.4) statistical computing environment. In the limma package, linear models were used to assess the differential expression in the designed microarray experiments. Genes with absolute fold change > 1.5, and *P* value <0.01 were considered differentially expressed genes (DEGs). Subsequently, pathway enrichment analysis was performed using the Ingenuity Pathway Analysis (IPA) tool. *P* values were calculated using Fisher's exact test, and *P* values <0.05 were considered significantly enriched in the canonical pathways [11].

### 2.10. Quantitative Real-Time PCR

Quantitative real-time PCR was performed to confirm the results of gene expression profiling. The RNA from retinas was extracted with the TRIzol reagent, and the RNA concentration of retinas was evaluated by Nanodrop2600 at 260 nm. Then, the RNA was reverse transcribed using a reverse transcription reagent kit (Roche, Basel, Switzerland) and 1 *μ*g RNA was inputted for the reaction. Real-time PCR was performed with SYBR Green PCR Mix (Roche, Basel, Switzerland) on an ABI Step One Plus® instrument (ABI, Carlsbad, California, USA). Reactions were performed in a volume of 10 *μ*L containing 2X SYBR Green PCR Mix, 5 *μ*L; cDNA, 1 *μ*L; forward and reverse primers, 0.4 *μ*L; and ddH2O, 3.6 *μ*L. *β*-Actin was served as the control. Differences in gene expression were quantified using the ΔΔCt method.

### 2.11. Western Blot

The retinas were added in ice-cold RIPA lysis buffer which contained a protease inhibitor mixture. The protein dissolved in supernatant was centrifuged and determined using the BCA protein assay reagent kit (Thermo Scientific, Waltham, MA, USA). SDS-PAGE gels containing 12% acrylamide were used to separate the samples (50 *μ*g). The proteins were transferred onto membrane, blocked for 1.5 h with 5% milk at 37°C, and then incubated with primary antibodies at 4°C overnight. Subsequently, the PVDF membranes were incubated with secondary antibody for 1.5 h. In the end, the bands were visualized using an electrochemiluminescence (ECL) kit and quantified using image analysis software.

### 2.12. Addition Statistical Analysis

One-way ANOVA with LSD or Tamhane test was used for comparison between the three groups. The statistical tests were performed using SPSS statistical processing software and *P* values less than 0.05 were considered statistically significant. The Kolmogorov-Smirnov test was used to confirm normal distribution. The results were presented as the means ± SEM, indicating the variability reliability of the data.

## 3. Results

### 3.1. HXJD Protects Retinal Structure in Rats with DR

**Figure 1:**
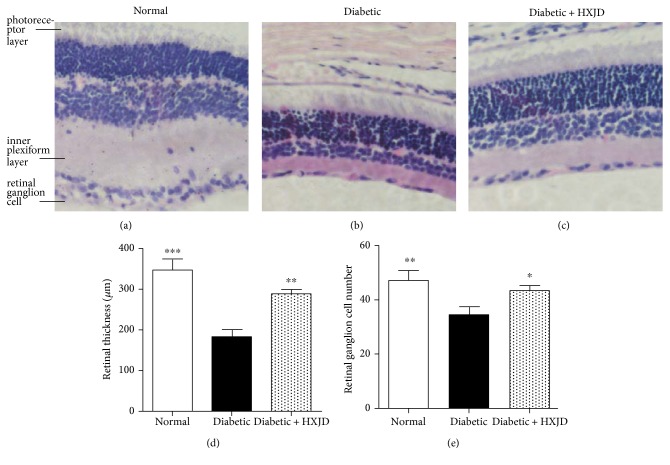
The effect of HXJD on retinal morphology. The retinas were stained with haematoxylin and eosin staining. (a) Retinas of normal rats. Retinal ganglion cell (RGC), inner plexiform layer (IPL), and photoreceptor layer (PL). (b) Retinas of diabetic rats. (c) Retinas of the rats in the HXJD group. Magnification: ×400. (d) The retinal thickness from IPL to PL in the midretina was measured. HXJD increased the retinal thickness. (e) The RGC number was counted. Retinas of the rats in the HXJD group showed an increased number of RGC. Data are represented as the means ± SEM. (*n* = 3~5). ^∗^*P* < 0.05, ^∗∗^*P* < 0.01, and ^∗∗∗^*P* < 0.001 compared with the diabetic group.

The HE staining was performed to observe the morphology of retinas. The retinas of the rats in normal group presented a clear structure, and the cells in every layer were uniformly distributed and densely packed. However, in the diabetic group, the retinas presented a reduction of cell density and oedema, and the cells were irregularly organized and loosely arranged. In addition, the retinal thickness and RGC number in the eyes of diabetic rats were decreased compared to those of normal rats (Figure 1). Expectedly, HXJD ameliorated the observed pathological changes. The retinal structure was improved, and the cells were organized with intact layers. Moreover, the retinal thickness and RGC number were evaluated in HXJD group (Figure 1).

### 3.2. HXJD Changes the Expression of Genes Associated with Cytokine Signalling and Matrix Metalloproteases

**Figure 2:**
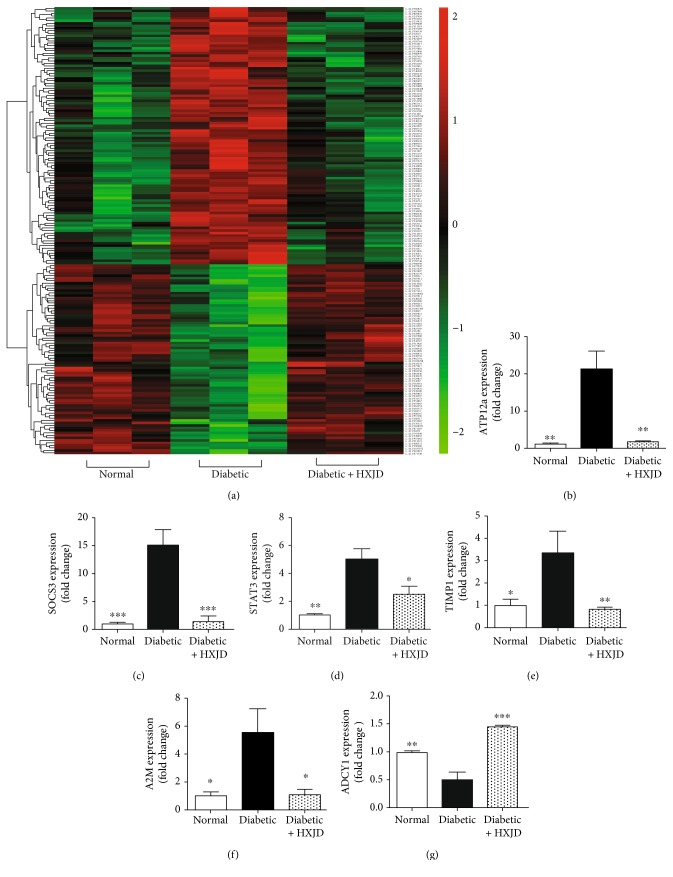
The effect of HXJD on the expression of genes associated with cytokine signalling and matrix metalloprotease expression. (a) Heat map of the regulated genes in normal, diabetic, and diabetic +HXJD rat groups. (b–g) The microarray data were validated using real-time PCR. Data are represented as the means ± SEM. (*n* = 3~4). ^∗^*P* < 0.05, ^∗∗^*P* < 0.01, and ^∗∗∗^*P* < 0.001, compared with the diabetic group.

A comparative analysis between the normal, diabetic, and HXJD groups for genome-scale gene expression was performed. The mRNA array chip included nearly forty thousand genes. Microarray analysis revealed that compared with the normal group, 215 genes were upregulated in the diabetic group, among which 99 genes were reversed in the HXJD group. In addition, 539 genes were downregulated in the diabetic group compared with the normal group, among which 71 genes were reversed in the HXJD group (Figure 2(a)).

To examine the function of differentially expressed mRNAs, IPA pathway annotation was applied. The results revealed that 37 canonical pathways were enriched within the 99 genes upregulated in diabetic group and subsequently downregulated after HXJD treatment, including the role of macrophages, fibroblasts, and endothelial cells in rheumatoid arthritis, matrix metalloprotease inhibition, and IL-6 signalling. Moreover, 18 canonical pathways were enriched within the 71 genes downregulated in the diabetic group and subsequently upregulated after HXJD treatment, which have been implicated in phototransduction pathways, the visual cycle, and RAR activation. The top 10 enriched pathways are shown, respectively, in Tables 1 and 2.

To verify the results of the microarray analysis, real-time PCR was performed to examine the levels of differentially expressed genes. ATP12A, which functions as a P-type cation transport ATPase [12], was highlighted as remarkably decreased after HXJD treatment. Microarray data demonstrated that ATP12A expression in the diabetic group was 34 times higher than the HXJD group, and PCR experiments achieved similar results (Figure 2(b)).

In addition, several genes associated with DR were selected for further study. SOCS3 and STAT3 are signal transducers involved in the regulation of cytokines [13, 14], including IL-6, IL-10, IL-22, and IL-9 (Table 1). TIMP1 and A2M govern the degradation of the extracellular matrix [15, 16]. The gene levels of SOCS3, STAT3, TIMP1, and A2M were increased in the retinas of diabetic rats compared to normal rat retinas, whereas these genes were downregulated in the retinas of HXJD-treated diabetic rats (Figures 2(c)–2(f)).

Moreover, ADCY1 was selected for PCR verification. This gene encodes a member of the of adenylate cyclase gene family [17]. ADCY1 expression was reduced in diabetic rats compared to normal rats, and the expression levels of this gene were reversed after treatment with HXJD (Figure 2(g)).

The PCR results demonstrated consistency between the microarray and real-time PCR data. This consistency indicated the reliability of the microarray results and confirmed the influence of HXJD on the expression of these genes.

### 3.3. HXJD Reduces the Protein Expression of p-STAT3, STAT3, SOCS3, A2M, and TIMP1

**Figure 3:**
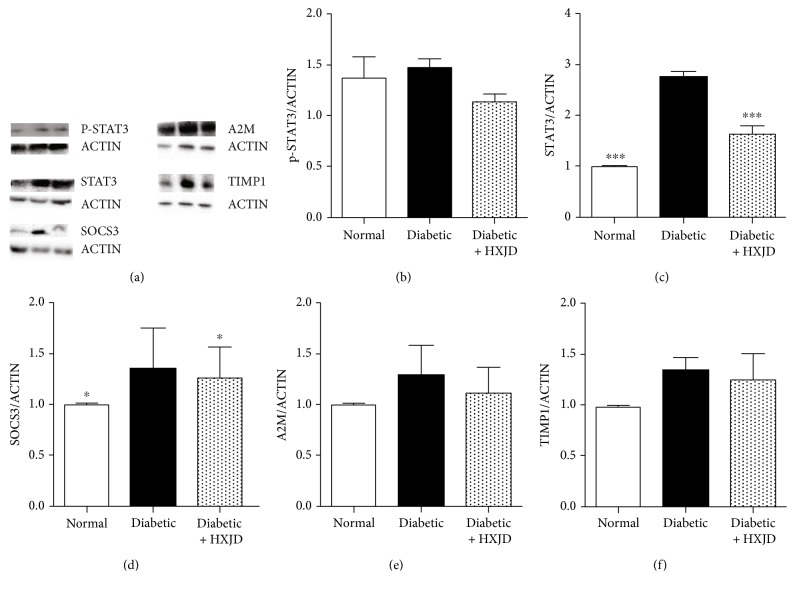
HXJD reduces the protein expression of p-STAT3, STAT3, SOCS3, A2M, and TIMP1 in the retinas of diabetic rats. The protein expression of p-STAT3, STAT3, SOCS3, A2M, and TIMP1 were measured using western blot. (a) Western blot images. (b–f) Image analysis of western blot. Data are represented as the means ± SEM. (*n* = 3). ^∗^*P* < 0.05 and ^∗∗∗^*P* < 0.001, compared with the diabetic group.

Western blot was carried out to examine the protein expression of p-STAT3, STAT3, SOCS3, A2M, and TIMP1 in the retinas of rats. As shown in Figure 3, in the diabetic rats, the protein levels of STAT3 and SOCS3 remarkably increased compared with the normal rats (Figures 3(c) and 3(d)). Meanwhile, HXJD reduced the protein expression of STAT3 and SOCS3 in the rat retinas (Figures 3(c) and 3(d)). Moreover, the protein levels of p-STAT3, A2M, and TIMP1 showed an increasing tendency in the diabetic group, compared with the normal group. And HXJD presented a decreasing tendency of p-STAT3, A2M, and TIMP1 (Figures 3(b), 3(e), and 3(f)).

### 3.4. HXJD Reduces the Expression of JAK2, p38 MAPK, and NF-*κ*B in the Retinas of Diabetic Rats

**Figure 4:**
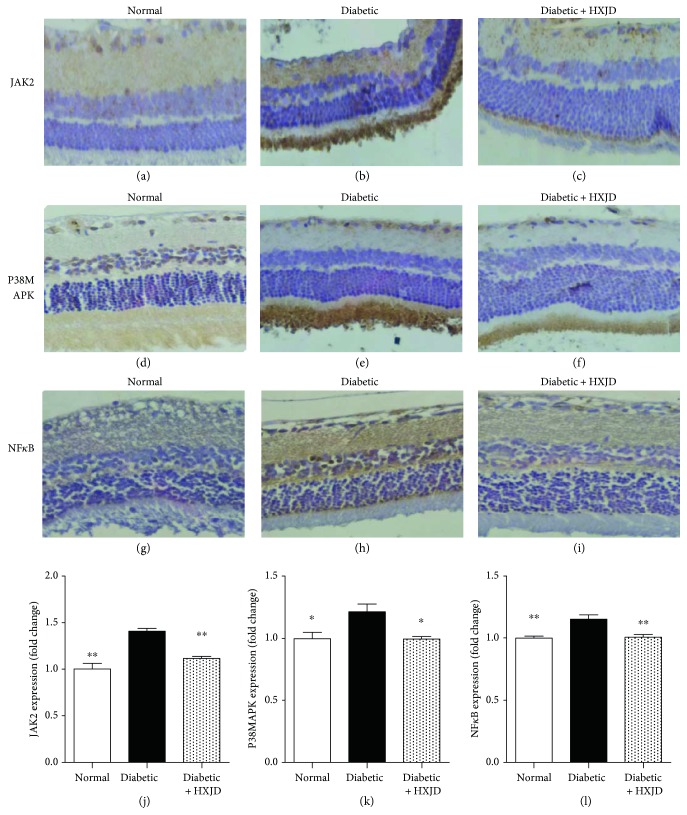
HXJD reduces the expression of JAK2, p38 MAPK, and NF-*κ*B in the retinas of diabetic rats. Distribution and expression of JAK2, p38 MAPK, and NF-*κ*B were measured using IHC. (a–c) IHC staining of JAK2. (d–f) IHC staining of p38 MAPK. (g–i) IHC staining of NF-*κ*B. Magnification: ×400. (j–l) Image analysis of JAK2, p38 MAPK, and NF-*κ*B. A reduction of JAK2, p38 MAPK, and NF-*κ*B was observed in the HXJD group. Data are represented as the means ± SEM. (*n* = 3~6). ^∗^*P* < 0.05 and ^∗∗^*P* < 0.01, compared with the diabetic group.

Immunohistochemistry was performed to evaluate the expression levels of JAK2, p38 MAPK, and NF-*κ*B in rat retinas. As shown in Figure 4, in the diabetic group, the expression of JAK2, p38 MAPK, and NF-*κ*B significantly increased in the retinas compared with that in the normal group (Figure 4). However, rats treated with HXJD presented the decreased expression of JAK2, p38 MAPK, and NF-*κ*B in the retinas (Figure 4).

## 4. Discussion

The aim of the present study was to clarify the mechanism of HXJD against DR. We used microarray technology to examine the retinal mRNA expression changes affected by HXJD. The results showed that 170 genes were altered after HXJD treatment, and these genes played critical roles in inflammation, proliferation, cytokine signalling, and retinal function. Among these processes, two mechanisms were verified: reduced SOCS3-STAT3 signalling pathway and inhibited TIMP1-A2M expression.

### 4.1. Reduced SOCS3-STAT3 Signalling Pathway

SOCS3 and STAT3 have been implicated in most of the pathways regulated by HXJD (Table 1). These proteins are signal transducers that govern essential cellular processes, such as proliferation, apoptosis, and inflammatory gene transcription. STAT3 mediates the signals from several receptor tyrosine kinases, including EGF, PDGF, IL-6, IL-10, and IL-12 [13, 18]. SOCS3 negatively regulates the STAT3 pathway [14]. In recent years, an increasing number of studies have demonstrated that SOCS3 and STAT3 mediate inflammation, apoptosis, and insulin signalling in hyperglycaemia-stimulated retinal endothelial cells [19]. Moreover, STAT3 is reported to regulate the expression of VEGF in various cells in different kinds of circumstances [20, 21]. The results of the present study showed that HXJD reduced SOCS3 and STAT3 levels in the retinas of diabetic rats. In addition, previous studies have shown that HXJD attenuated retinal apoptosis and VEGF expression in diabetic rats [5]. Thus, we speculated that HXJD might ameliorate hyperglycaemia-induced apoptosis and VEGF in the retinas of diabetic rats through controlling SOCS3 or STAT3 expression.

Since SOCS3-STAT3 signal transduction is fundamental for a wide variety of physiological processes and pathological status, we further evaluated the proteins involved in SOCS3-STAT3 pathway. JAK2 activates and phosphorylates STAT3 [22]. In addition, NF-*κ*B [23, 24] and p38 MAPK [25] promote SOCS3-STAT3 signalling. HXJD decreased the levels of JAK2, p38 MAPK, and NF-*κ*B in the retinas of diabetic rats, suggesting that HXJD may arrest the progression of DR through the inhibition of the SOCS3-STAT3 pathway.

In addition to clarifying the pharmacological mechanisms of HXJD in preventing DR, the present study enhances the current understanding of the factors underlying the development of DR. In the early-middle stages of diabetes, the levels of STAT3 and SOCS3 in the retinas are increased [26–28]. Here, we provided new evidence that SOCS3 and STAT3 gene expression were also elevated in the late period of diabetes, as the present study was conducted for 32 weeks. Moreover, the results revealed that other key members of the SOCS3-STAT3 pathway were also disturbed in the diabetic retina. Taken together, these results suggest that the SOCS3-STAT3 signalling pathway participates in the development of DR. Furthermore, these results emphasize the necessity to assess the levels of SOCS3 and STAT3 in patients with DR. Thus, SOCS3 and STAT3 may represent new markers in the process of DR.

Since the SOCS3-STAT3 pathway plays a fundamental role in the pathogenesis of DR, it is necessary to explore new type of drugs for the treatment of DR by targeting SOCS3 or STAT3. Some drugs used to treat diabetes or DR, such as statins [27], enalapril [29], and tazones [30], decrease SOCS3 and STAT3 expression in diabetic animals. However, until recently, there are no drugs in clinical use that specifically target SOCS3-STAT3. We propose the exploration of a new active compound, HXJD, which targets SOCS3 or STAT3. In previous studies, we identified seven compounds in the active fractions of HXJD, including notoginsenoside R4, ginsenoside Ra3, ginsenoside Rb1, 20S-ginsenoside Rg2, ginsenoside Re, ginsenoside Rd, and notoginsenoside R1 [31]. And this time, we examined the content of rutin, berberine and ginsenoside Rg1 in HXJD. It has been reported berberine from *C. chinensis* notably attenuates the excessive expression of p-STAT3 in the rats with autoimmune myocarditis [32]. Ginsenoside Rb1 [33], from *P. notoginseng*, has been reported to decrease the levels of p-STAT3 and SOCS3 in ovarian cancer cells, microglial cells, and obese mice. Berberine and ginsenoside Rb1 may be among the active compounds of HXJD. However, until recently, studies supporting a role for the SOCS3-STAT3 pathway in the effect of HXJD are only correlative, as studies to determine the action of HXJD by directly blocking SOCS3-STAT3 signalling molecules are more rigorous.

### 4.2. Inhibited TIMP1-A2M Expression

The inhibition of matrix metalloproteases (MMPs) showed the second highest score among the HXJD-downregulated pathways (Table 1). TIMP1 belongs to a family of proteinase inhibitors that regulate matrix metalloproteinase activity by forming one-to-one stoichiometric complexes [15]. TIMP1 can irreversibly inactivate substrates, such as MMP2 and MMP9 [34], but the final response is dependent on the ratio of TIMPs/MMPs rather than the separate levels of TIMPs and MMPs [35]. In addition, TIMP1 shows protease-independent regulatory activities [36]. A2M is a broad-spectrum protease inhibitor that regulates the activities of MMPs [16]. Studies have demonstrated that TIMP1 and A2M expression is increased in the eyes of patients or animals with diabetic retinopathy [37, 38]. Thus, the TIMP1-A2M pathway likely plays important roles in the pathological processes of DR. In the present study, HXJD inhibited the dysregulated expression of TIMP1 and A2M genes. However, the gene levels of MMP2/9 showed no significant differences between the three groups analyzed. In a previous experiment, we observed that HXJD reduced the protein content of MMP2/9 in the retinas of diabetic rats [7], likely reflecting the fact that HXJD effects the posttranslation of MMP2/9. Taken together, these results suggested that HXJD might impede the progress of DR through the inhibition of the TIMP1-A2M pathway.

## 5. Conclusions

**Figure 5:**
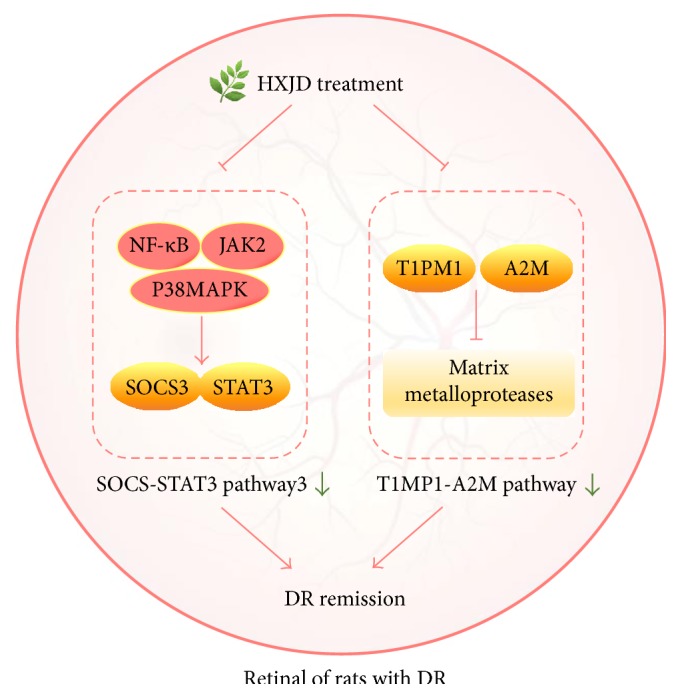
The molecular mechanism of HDJX in attenuating diabetic retinopathy in rats via the inhibition of SOCS3-STAT3 and T1MP1-A2M pathways. JAK2, NF-*κ*B, and p38 MAPK promote SOCS3-STAT3 signalling, then SOCS3-STAT3 is involved in the progression of DR. TIMP1 and A2M play roles in matrix degradation which also take part in the process of DR. HXJD has the ability of inhibiting these pathological changes and thereafter it will ameliorate DR.

HXJD ameliorated retinal structure and pathological changes in DR. Moreover, the microarray data showed that HXJD reduced the expression of SOCS3 and STAT3 and decreased the levels of TIMP1 and A2M expression. In conclusion, the protective role of HXJD against DR is likely achieved by affecting SOCS3-STAT3 and TIMP1-A2M pathways (Figure 5). These findings lay the foundation for the active research and therapeutic application of HXJD.

## Figures and Tables

**Table 1:** Top 10 canonical pathways identified using IPA (downregulated by HXJD).

Ingenuity canonical pathways	*P* value	Affected molecules
Role of macrophages, fibroblasts, and endothelial cells in rheumatoid arthritis	1.15*E*−04	TLR2, IL1R2, SOCS3, WNT10A, STAT3, IL17RA, and IRAK2
Inhibition of matrix metalloproteases	4.07*E*−04	MMP7, TIMP1, and A2M
IL-6 signalling	9.12*E*−04	IL1R2, SOCS3, STAT3, and A2M
IL-10 signalling	2.04*E*−03	IL1R2, SOCS3, and STAT3
Growth hormone signalling	2.14*E*−03	SOCS3, STAT3, and A2M
IL-22 signalling	3.55*E*−03	SOCS3, STAT3
Role of JAK family kinases in IL-6-type cytokine signalling	3.89*E*−03	SOCS3, STAT3
IL-9 signalling	6.31*E*−03	SOCS3, STAT3
Role of JAK2 in hormone-like cytokine signalling	7.41*E*−03	SOCS3, STAT3
Tetrahydrobiopterin biosynthesis I	1.10*E*−02	GCH1

**Table 2:** Top 10 canonical pathways identified using IPA (upregulated by HXJD).

Ingenuity canonical pathways	*P* value	Affected molecules
Phototransduction pathway	2.57*E*−04	ARR3, GRK1, and PDE6D
The visual cycle	5.50*E*−04	RDH11, RDH12
RAR activation	1.00*E*−03	RDH11, ADCY1, RDH12, and CITED2
Retinoate biosynthesis I	2.69*E*−03	RDH11, RDH12
Retinol biosynthesis	2.69*E*−03	RDH11, RDH12
Creatine-phosphate biosynthesis	1.17*E*−02	CKMT1A/CKMT1B
Protein kinase A signalling	1.26*E*−02	H3F3A/H3F3B, ADCY1, GRK1, and PDE6D
NAD biosynthesis III	1.26*E*−02	Nmnat3
NAD biosynthesis from 2-amino-3-carboxymuconate semialdehyde	1.62*E*−02	Nmnat3
NAD salvage pathway III	1.62*E*−02	Nmnat3

## Abbreviations

DR: Diabetic retinopathy
SOCS: Suppressor of cytokine signalling
STAT: Signal transducer and activator of transcription
TIMP: Tissue inhibitor matrix metalloproteinase
A2M: a2-macroglobulin
VEGF: Vascular endothelial growth factor
MMP: Matrix metalloproteinase
STZ: Streptozocin
ADCY1: Type 1 adenylyl cyclase
JAK: Janus kinase
NF-*κ*B: Nuclear factor-kappa B
MAPK: Mitogen-activated protein kinase
ONL: Outer nuclear layer
INL: Inner nuclear layer
RGC: Retinal ganglion cells.
